# Novel Highly Pathogenic Avian Influenza A(H5N1) Clade 2.3.4.4b Virus in Wild Birds, South Korea

**DOI:** 10.3201/eid2907.221893

**Published:** 2023-07

**Authors:** Sun-hak Lee, Andrew Y. Cho, Tae-hyeon Kim, Seo-jeong Ahn, Ju Ho Song, Heesu Lee, Yun-Jeong Choi, Nyamsuren Otgontogtokh, Jung-Hoon Kwon, Chang-Seon Song, Dong-Hun Lee

**Affiliations:** Author affiliations: Konkuk University, Seoul, South Korea (S.-h. Lee, A.Y. Cho, T.-h. Kim, S.-j. Ahn, J.H. Song, H. Lee, Y.-J. Choi, N. Otgontogtokh, C.-S. Song, D.-H. Lee); Kyungpook National University, Daegu, South Korea (J.-H. Kwon)

**Keywords:** influenza, highly pathogenic avian influenza virus, H5N1, wild birds, phylogenetic analysis, respiratory infections, viruses, zoonoses, South Korea, clade 2.3.4.4b

## Abstract

We isolated 5 highly pathogenic avian influenza A(H5N1) clade 2.3.4.4.b viruses from wild waterfowl feces in South Korea during November 2022. Whole-genome sequencing and phylogenetic analysis revealed novel genotypes produced by reassortment with Eurasian low pathogenicity avian influenza viruses. Enhanced surveillance will be required to improve prevention and control strategies.

Highly pathogenic avian influenza viruses (HPAIVs) have caused major economic losses in the poultry industry and are a major threat to public health. Since the first detection of HPAIV A(H5N1) from a goose in 1996 in Guangdong, China, its descendants have evolved into multiple hemagglutinin (HA) gene-specific clades (H0–H9) and subclades (*1*) causing intercontinental epizootics (*2*). Over several decades, H5 HPAIVs have evolved into multiple subtypes and genotypes generated by reassortment with low pathogenicity avian influenza viruses (LPAIVs), which led to emergence of clade 2.3.4.4 H5Nx HPAIVs in eastern China during 2013–2014 (*3*).

In mid-2016, reassortant H5N8 clade 2.3.4.4b HPAIVs that contained internal genes of LPAIVs from Eurasia were detected in wild birds at Uvs-Nuur Lake in Russia and Qinghai Lake in China (*4*); the viruses caused large outbreaks in Europe during 2016–2017 (*5*). Subsequently, various novel reassortant H5N8 HPAIVs were detected in Eurasia (*5*,*6*). In late 2020, novel reassortant clade 2.3.4.4b H5N1 HPAIVs were detected and became predominant in Europe in poultry and wild birds (*5*). 

We isolated 5 H5N1 HPAIVs from wild bird feces collected in South Korea in November 2022 (Appendix 1): A/Spot-billed_duck/Korea/K22-730-1/2022(H5N1) [K22-730-1], A/Wild_bird/Korea/K22-742/2022(H5N1) [K22-742], A/Spot-billed_duck/Korea/K22-856-2/2022(H5N1) [K22-856-2], A/Spot-billed_duck/Korea/K22-862-1/2022(H5N1) [K22-862-1], and A/Spot-billed_duck/Korea/K22-920/2022(H5N1) [K22-920] (Appendix 1 Table 1). To rapidly share the information, we conducted whole-genome sequencing of the isolates and deposited the genome sequences in the GISAID database (https://www.gisaid.org).

All H5N1 isolates were classified as HPAIV on the basis of HA cleavage site amino acid sequences (PLRPKRRKR/G). The 5 isolates shared high nucleotide sequence identities (99.4% to ≈100%) across all 8 influenza genes, except for the K22-920 isolate polymerase basic protein 1 (PB1), polymerase acidic protein (PA), nucleoprotein (NP), and nonstructural (NS) genes (93.0% to ≈99.0%). BLAST (https://blast.ncbi.nlm.nih.gov) search results showed HA, neuraminidase, and matrix (M) protein genes of all isolates had >99.1% identities with 2021–2022 clade 2.3.4.4b HPAIVs (Table 1). PB1, PA, NP, and NS genes of all isolates were highly similar (98.72%–99.52%) to 2019–2022 LPAIVs from East Asia. PB1, PA, NP, and NS genes of K22-920 were similar to 2019–2020 LPAIVs from South Korea, Russia, and Bangladesh (>98.4%–99.3%).

**Table 1:** Nucleotide sequence identities between gene segments of 5 novel clade 2.3.4.4b highly pathogenic avian influenza A(H5N1) viruses from wild birds in South Korea and nearest homologs in the GISAID Epiflu database*

Isolates	Gene	Virus	Accession no.†	% Identity
All 5 isolates	PB2	A/goose/Hunan/SE284/2022(H5N1)/HPAI	EPI2029895	99.21%
		A/duck/Mongolia/826/2019 (H4N6)/LPAI	EPI1777578	98.33%
	HA	A/turkey/Tyumen/81-96V/2021 (H5N1)/HPAI	EPI1958105	99.47%
		A/chicken/Tyumen/47-79V/2021 (H5N1)/HPAI	EPI1957985	99.41%
	NA	A/goose/Hunan/SE284/2022 (H5N1)/HPAI	EPI2029897	99.22%
		A/duck/Bangladesh/51600/2021 (H5N1)/HPAI	EPI2163444	99.15%
	M	A/ibis/Egypt/RLQP-229S/2022 (H5N1)/HPAI	EPI2201158	99.80%
		A/duck/Bangladesh/19D1817/2021 (H5N1)/HPAI	EPI2062119	99.80%
K22-730-1, K22-742, K22-856-2, K22-862-1‡	PB1	A/Mallard/South Korea/KNU2019-20/2019 (H5N1)/LPAI	EPI1902594	98.77%
		A/duck/Saga/411117/2013 (H6N1)/LPAI	EPI855573	98.72%
	PA	A/duck/Bangladesh/18D1811/2022 (H5N3)/LPAI	EPI1997269	99.44%
		A/duck/Bangladesh/17D1839/2022 (H5N3)/LPAI	EPI1997245	99.44%
	NP	A/Northern Shoveler/South Korea/KNU2021-13/2021 (H11N9)/LPAI	EPI2153438	99.40%
		A/Mallard/South Korea/KNU2019-51/2019 (H5N3)/LPAI	EPI1902887	99.33%
	NS	A/mallard/Yakutia/47/2020 (H7N7)/LPAI	EPI1848197	99.52%
		A/Wild duck/South Korea/KNU2020-31/2020 (H1N1)/LPAI	EPI1931606	99.40%
K22-920§	PB1	A/mallard/Anhui/3-617/2019 (H6N1)/LPAI	EPI1743666	98.46%
		A/Eurasian_Curlew/China/CZ322(7)/2019 (H3N8)/LPAI	EPI1890551	98.46%
	PA	A/common teal/Amur region/92b/2020 (H6N2)/LPAI	EPI1849993	99.26%
		A/mallard/Russia Primorje/94T/2020 (H1N1)/LPAI	EPI1849961	99.21%
	NP	A/Spot-billed duck/South Korea/KNU2020-105/2020 (H3N2)/LPAI	EPI1931651	99.33%
		A/Eurasian teal/South Korea/JB32-15/2019 (H10N7)/LPAI	EPI1903752	98.80%
	NS	A/mallard/Yakutia/47/2020 (H7N7)/LPAI	EPI1848197	99.28%
		A/duck/Bangladesh/39397/2019 (H10N3)/LPAI	EPI1778261	99.28%

*HPAI influenza viruses isolated from wild bird feces in November 2022. HA, hemagglutinin; HPAI, highly pathogenic avian influenza; LPAI, low pathogenicity avian influenza; M, matrix protein; NP, nucleoprotein; NS, nonstructural; PA, polymerase acidic; PB, polymerase basic. †Accession numbers of nearest homologs as of December 8, 2022, from GISAID (https://www.gisaid.org). ‡Genotype I. §Genotype II.

In maximum-likelihood phylogenetic analyses, PB2, HA, neuraminidase, and M genes of the 5 H5N1 viruses from South Korea clustered with those of viruses previously described as genotype G10, identified in China during 2022–2023 (Appendix 1 Figures 1–8); G10 is a natural reassortant H5N1 HPAIV containing the PB2 gene from LPAIVs (*7*). PB1, PA, NP, and NS genes of all H5N1 viruses from South Korea except K22-920 clustered with those of LPAIVs from Asia; those gene segments in K22-920 clustered separately with other LPAIVs from Asia, including South Korea, Russia, and Bangladesh (Appendix 1 Figures 2, 3, 5, 8). Bayesian phylogeny of the HA gene indicated the H5N1 viruses from South Korea formed a well-supported cluster; time to most recent common ancestor was estimated to be August 11, 2022 (95% highest posterior density June 11–October 11, 2022), suggesting those H5N1 HPAIVs most likely emerged 1–2 months before the autumn wild bird migration to South Korea (Figure 1; Appendix 1 Figure 9). The isolates from South Korea shared recent common ancestry with the A/Jiangsu/NJ210/2023(H5N1) virus; time to most recent common ancestor between them was April 12, 2022 (95% highest posterior density December 26, 2021–July 28, 2022), suggesting the ancestral H5N1 HPAIVs had been circulating undetected for ≈7 months.

**Figure 1:**
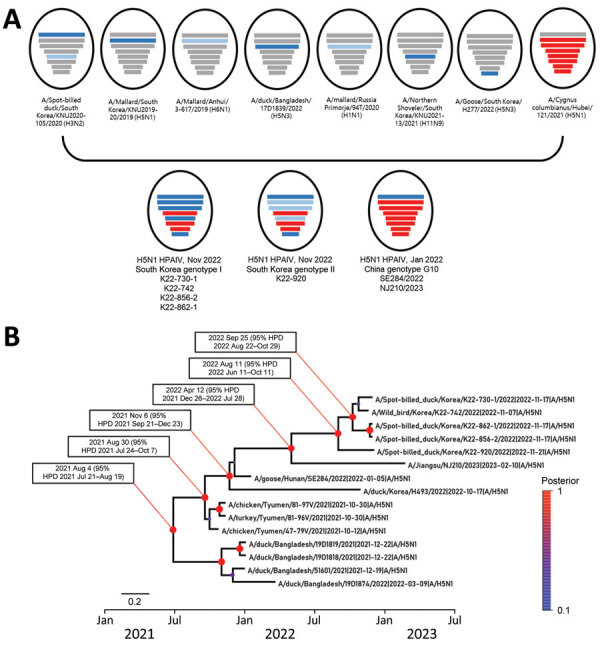
Phylogenetic analysis of novel highly pathogenic avian influenza A(H5N1) clade 2.3.4.4b viruses found in wild bird feces in South Korea, November 2022. A) Schematic representation of origin of virus isolates from South Korea compared with genotype G10 viruses found in China. Bars represent 8 gene segments of avian influenza virus in the following order (top to bottom): polymerase basic 2, polymerase basic 1, polymerase acidic, hemagglutinin, nucleoprotein, neuraminidase, matrix, and nonstructural. Different bar colors indicate different virus origins estimated from maximum-likelihood phylogenetic trees. Gene segments originating from highly pathogenic avian influenza viruses are indicated by red bars. Gene segments originating from low pathogenicity avian influenza viruses are indicated by blue bars. B) Time-scaled maximum clade credibility tree for hemagglutinin gene segments from novel viruses isolated in South Korea (5 viruses at top). Red to blue colored scale on right side indicates posterior clade probabilities at nodes. Scale bar indicates nucleotide substitutions per site. HPD, highest posterior density.

The H5N1 HPAIVs from South Korea contained amino acids in HA with binding affinity for avian α-2,3-linked sialic acid receptors (T118, V210, Q222, and G224) (H5 numbering) (*8*,*9*). They also had 2 HA amino acid substitutions, S113A and T156A, associated with increased binding affinity to human α-2,6-linked sialic acid receptors (Appendix 1 Table 2). All 5 isolates had amino acid substitutions that included A515T in PA, known to increase polymerase activity in mammal cells, and N30D, I43M, T215A in MP1 and L89V in PB2, known to increase virulence in mice (Appendix 1 Tables 2, 3).

The HPAI/LPAI reassortment of H5Nx clade 2.3.4.4b HPAIVs created a diverse genetic pool of H5 clade 2.3.4.4 viruses that continuously emerged in various countries (*1*). Clade 2.3.4.4 H5N8 HPAIV isolated from Uvs-Nuur Lake in Russia had reassorted H3N8 LPAIV genes from Mongolia (*4*). In Europe, HPAIVs identified in 2020 (*5*,*6*) were produced by reassortment between clade 2.3.4.4b HPAIV and LPAIVs from Eurasia. Novel reassortments of clade 2.3.4.4 HPAIV and LPAIVs from Eurasia were also detected in 2016 (*10*), during 2020–2021 (Appendix 1 reference *1*), and in late 2021 (Appendix 1 reference *2*) in South Korea. Considering the continuous emergence and global dissemination of novel reassortant clade 2.3.4.4b HPAI H5Nx viruses, enhanced active surveillance in wild animals and domestic poultry will be required to monitor the introduction, dissemination, and evolution of HPAIVs and provide insight for improved prevention and control strategies.

Appendix 1 Additional information for novel clade 2.3.4.4b highly pathogenic avian influenza A(H5N1) virus in wild birds, South Korea.

Appendix 2 GISAID data used in investigation of novel clade 2.3.4.4b highly pathogenic avian influenza A(H5N1) virus in wild birds, South Korea.
