# Correction: Vol. 29, No. 7

**DOI:** 10.3201/eid3007.C13007

**Published:** 2024-07

**Keywords:** errata, erratum, correction

Funding information was missing in Novel Highly Pathogenic Avian Influenza A(H5N1) Clade 2.3.4.4b Virus in Wild Birds, South Korea (S.-h. Lee et al.). The article has been corrected online (https://wwwnc.cdc.gov/eid/article/29/7/22-1893_article).

