# Supplementary material for: Novel Highly Pathogenic Avian Influenza A(H5N1) Clade 2.3.4.4b Virus in Wild Birds, South Korea
Source: Emerg Infect Dis. 2023 Jul;29(7):1475–8. doi: 10.3201/eid2907.221893 (PMC10310373; doi:10.3201/eid2907.221893)
Supplement: Appendix 1 — Additional information for novel clade 2.3.4.4b highly pathogenic avian influenza A(H5N1) virus in wild birds, South Korea. [file 22-1893-Techapp-s1.pdf]

Article DOI: https://doi.org/10.3201/eid2907.221893
EID cannot ensure accessibility for supplementary materials supplied by authors. Readers who have difficulty accessing supplementary content should contact the authors for assistance.

Novel Highly Pathogenic Avian Influenza A(H5N1) Clade 2.3.4.4b Virus in Wild Birds, South Korea

Appendix 1

Additional Methods

Virus Detection and Isolation

A total of 3,490 wild waterfowl fecal samples were collected during September 8–November 23, 2022. Only fresh fecal samples in wild bird habitats, including Gokgyo stream, Anseong stream, and Miho River in South Korea, were collected for routine highly pathogenic avian influenza A virus (HPAIV) surveillance. Those sites were selected because numerous avian influenza viruses have been identified previously, including H5Nx HPAIVs (*3*). The fecal samples were resuspended in phosphate-buffered saline containing 400 mg/mL gentamycin and then clarified by centrifugation (3,000 rpm for 10 min at 4°C). Supernatants were filtered through a 0.45 μm syringe filter and used to inoculate 10-day-old specific pathogen-free embryonated chicken eggs for virus isolation. Harvested allantoic fluids were tested for hemagglutinin (HA) activity. Total RNA was extracted from allantoic fluids positive for HA by using the RNeasy mini kit (QIAGEN, https://www.qiagen.com) according to the manufacturer's instructions. RNA was tested for H5 and H7 subtypes and influenza A virus matrix protein gene by real-time reverse transcription PCR as previously described (*4*). Influenza A–positive and H5-positive samples were detected in 3 of 30 collection trips (Appendix 1 Table 1). Host species for the viruses were identified by DNA barcoding the cytochrome c oxidase subunit 1 gene as previously described (*5*).

Whole-Genome Sequencing and Assembly

All 8 virus genes from each isolate were amplified by using the OneTaq 2× Master Mix (New England BioLabs, https://www.neb.com) and universal primers as previously described

(*6*). Next generation sequencing was performed on an iSeq100 instrument (Illumina, https://www.illumina.com). Library preparation was performed by using the Illumina DNA library prep kit. Whole genome sequences were assembled by using the Iterative Refinement Meta-Assembler (https://wonder.cdc.gov/amd/flu/irma) module and an in-house shell script. Assembly was visualized by using Geneious Prime software (https://www.geneious.com). Assembled genomes were uploaded in the GISAID EpiFlu database (https://www.gisaid.org) under accession nos. EPI_15943002, EPI_15943015, EPI_15944663, EPI_15944665, EPI_15944667) (Appendix 1 Table 1)

Phylogenetic Inference

We conducted BLAST (https://blast.ncbi.nlm.nih.gov) searches in the GISAID Epiflu database and retrieved the top 100 hits for comparative phylogenetic analysis. We also used genome sequences of representative HPAI viruses that have been identified in Asia, Europe, and North America since 2021. Complete coding regions were aligned by using MAFFT (https://mafft.cbrc.jp/alignment/software). We generated maximum-likelihood trees of each gene segment by using the RAxML program and general time-reversible plus gamma nucleotide substitution model with 1,000 rapid bootstrap replicates (*7*) (Appendix 1 Figures 1–8). We performed Bayesian relaxed-clock phylogenetic analysis of HA genes by using BEAST version 1.10.4 (https://beast.community) and applied an uncorrelated log-normal distribution relaxed-clock method; the Hasegawa, Kishino, and Yano plus gamma nucleotide substitution model; and Gaussian Markov random field Bayesian Skyride coalescent prior method (Appendix 1 Figure 9). A Markov chain Monte Carlo method (*8*) to sample trees and evolutionary parameters was run for 50–100 million generations. Independent chains (≥3) were combined for adequate sampling of the posterior distribution of the trees. The output data were analyzed by using TRACER v1.4 (https://beast.bio.ed.ac.uk/tracer) with 5% burn-in. A maximum clade credibility tree was generated by using TreeAnnotator in BEAST and visualized with FigTree v1.4.2 (https://tree.bio.ed.ac.uk).

References

1. Baek YG, Lee YN, Lee DH, Shin JI, Lee JH, Chung DH, et al. Multiple reassortants of H5N8 clade 2.3.4.4b highly pathogenic avian influenza viruses detected in South Korea during the winter of 2020–2021. Viruses. 2021;13:490. PubMed https://doi.org/10.3390/v13030490

2. Sagong M, Lee YN, Song S, Cha RM, Lee EK, Kang YM, et al. Emergence of clade 2.3.4.4b novel reassortant H5N1 high pathogenicity avian influenza virus in South Korea during late 2021. Transbound Emerg Dis. 2022;69:e3255–60. PubMed https://doi.org/10.1111/tbed.14551

3. Cui P, Shi J, Wang C, Zhang Y, Xing X, Kong H, et al. Global dissemination of H5N1 influenza viruses bearing the clade 2.3.4.4b HA gene and biologic analysis of the ones detected in China. Emerg Microbes Infect. 2022;11:1693–704. PubMed https://doi.org/10.1080/22221751.2022.2088407

4. Spackman E, Senne DA, Bulaga LL, Myers TJ, Perdue ML, Garber LP, et al. Development of real-time RT-PCR for the detection of avian influenza virus. Avian Dis. 2003;47:1079–82. PubMed https://doi.org/10.1637/0005-2086-47.s3.1079

5. Lee DH, Lee HJ, Lee YJ, Kang HM, Jeong OM, Kim MC, et al. DNA barcoding techniques for avian influenza virus surveillance in migratory bird habitats. J Wildl Dis. 2010;46:649–54. PubMed https://doi.org/10.7589/0090-3558-46.2.649

6. Lee DH. Complete genome sequencing of influenza A viruses using next-generation sequencing. Methods Mol Biol. 2020;2123:69–79. PubMed https://doi.org/10.1007/978-1-0716-0346-8_6

7. Stamatakis A. RAxML version 8: a tool for phylogenetic analysis and post-analysis of large phylogenies. Bioinformatics. 2014;30:1312–3. PubMed https://doi.org/10.1093/bioinformatics/btu033

8. Suchard MA, Weiss RE, Sinsheimer JS. Bayesian selection of continuous-time Markov chain evolutionary models. Mol Biol Evol. 2001;18:1001–13. PubMed https://doi.org/10.1093/oxfordjournals.molbev.a003872

Appendix 1 Table 1. Clade 2.3.4.4b H5N1 highly pathogenic avian influenza viruses isolated in this study.

Identification	Subtype	Collection Date	Host species*	Collection location	Latitude	Longitude	GISAID no.
A/Spot-billed_duck/Korea/K22–730–1/2022	H5N1	2022 Nov 17	Anas poecilorhyncha	Asan-si, Gokgyo stream	36.79785	127.0072	EPI_15943002
A/Wild_bird/Korea/K22–742/2022	H5N1	2022 Nov 07	NI	Asan-si, Gokgyo stream	36.79785	127.0072	EPI_15943015
A/Spot-billed_duck/Korea/K22–856–2/2022	H5N1	2022 Nov 17	Anas poecilorhyncha	Anseong-si, Anseong stream	36.99126	127.2079	EPI_15944663
A/Spot-billed_duck/Korea/K22–862–1/2022	H5N1	2022 Nov 17	Anas poecilorhyncha	Anseong-si, Anseong stream	36.99126	127.2079	EPI_15944665
A/Spot-billed_duck/Korea/K22–920/2022	H5N1	2022 Nov 21	Anas poecilorhyncha	Cheongju-si, Miho River	36.65164	127.3781	EPI_15944667

*Host species were identified by DNA Barcoding of mitochondrial DNA. NI, not identified.

Appendix 1 Table 2. Amino acid substitutions in hemagglutinin and polymerase basic 2 proteins from clade 2.3.4.4b H5N1 highly pathogenic avian influenza viruses isolated in South Korea, November 2022*

	Hemagglutinin†										Polymerase basic protein 2‡				
Isolate	D94N	S123A	126	S133A	S154N	T156A	T188I	V210I	Q222L	G224S	L89V	D256G	Q591K	E627K	D701N
Genotype I	S	P	E	A	D	A	T	V	Q	G	V	D	Q	E	D
Genotype II	S	P	E	A	D	A	T	V	Q	G	V	D	Q	E	D
Hunan/SE284	S	P	E	A	D	A	T	V	Q	G	V	D	Q	E	D

*Genotype I contained isolates K22–730–1, K22–742, K22–856–2, and K22–862–1 from South Korea. Genotype II contained isolate K22–920 from South Korea. Hunan/SE284 was genotype G10 isolated from China.

†Hemagglutinin H5 subtype numbering was used. D94N, S123P, S133A, S154N, T156A, T188I, Q222L, and G224S mutations in hemagglutinin are associated with increased binding to human α-2,6 sialic acid receptors.

‡L89V and D256G mutations in PB2 are known to be associated with increased virulence in mice. Q591K, E627K, and D701N mutations in PB2 are associated with increased viral replication in mammals.

Appendix 1 Table 3 Amino acid substitutions in polymerase acidic, matrix, and nonstructural proteins from clade 2.3.4.4b H5N1 highly pathogenic avian influenza viruses isolated in South Korea, November 2022*

	PA†	Matrix protein 1‡			Nonstructural protein§				
Isolate	A515T	N30D	I43M	T215A	P42S	Δ80–84	L98F	I101M	ESEV
Genotype I	T	D	M	A	A	AIASS	I	E	ESEV
Genotype II	T	D	M	A	A	AIASS	I	E	ESEV
Hunan/SE284	T	D	M	A	S	TIAPV	M	D	ESEV

*Genotype I contained isolates K22–730–1, K22–742, K22–856–2, and K22–862–1 from South Korea. Genotype II contained isolate K22–920 from South Korea. Hunan/SE284 was genotype G10 isolated from China. PA, polymerase acidic protein.

†A515T mutation in PA is known to be associated with avian influenza subtype H5 transmissibility in ferrets.

‡N30D, I43M, and T215A mutations in matrix protein 1 are known to be associated with increased virulence in mice.

§P42S mutation, 80–84 deletion, and ESEV PDZ-binding motif mutations in nonstructural protein are known to be associated with increased virulence in mice.

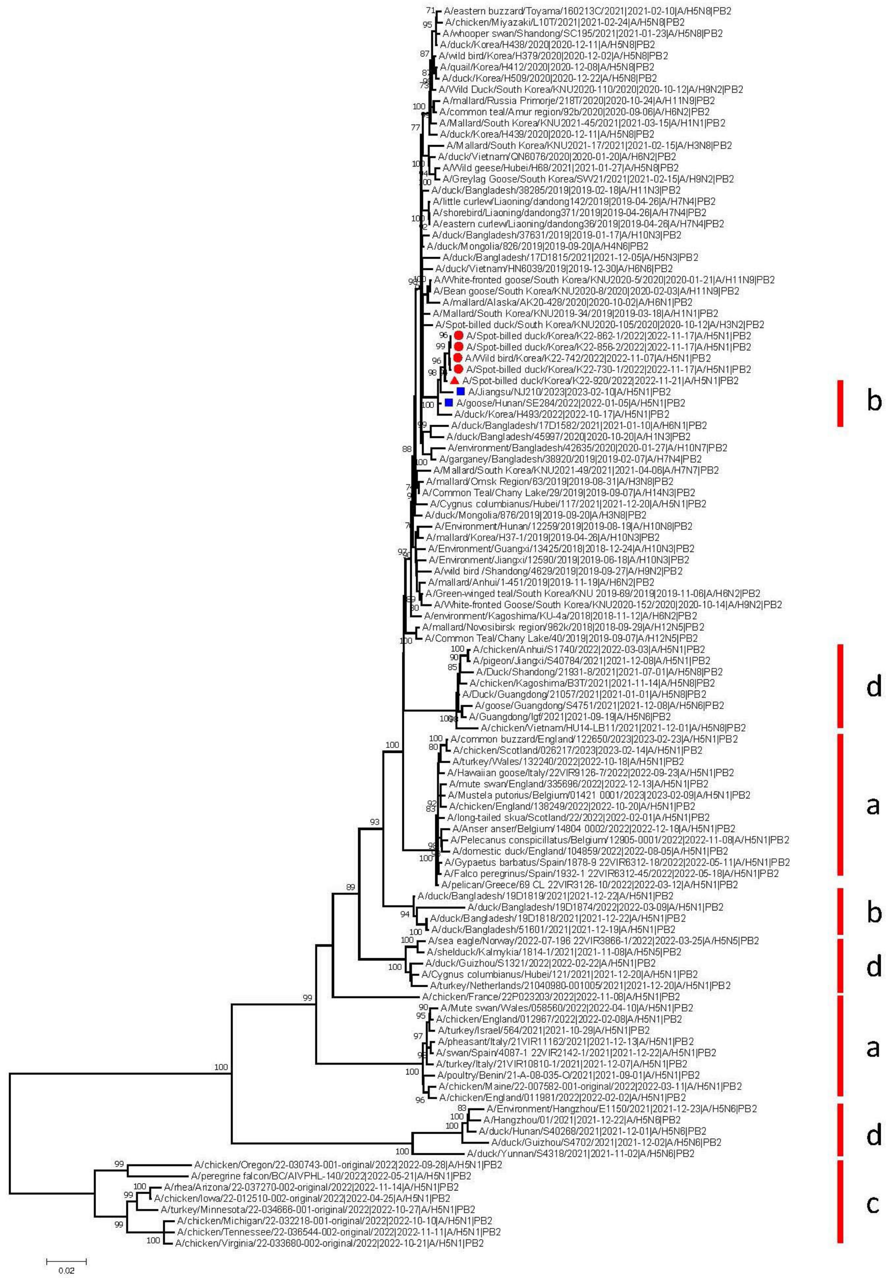

Appendix 1 Figure 1. Maximum-likelihood phylogenetic tree of polymerase basic 2 gene segment from avian influenza viruses. Bootstrap values >70% are shown. Highly pathogenic avian influenza virus isolates K22–862–1, K22–856–2, K22–742, K22–730–1 from South Korea are indicated by solid red circles, K22–920 from South Korea by solid red triangle, and genotype G10 isolates A/Hunan/SE284/2022 and A/Jiangsu/NJ10/2023 from China by blue rectangles. Red vertical lines indicate gene clusters mainly detected in Europe (a), Asia, including Bangladesh, Russia, and China (b), North America (c), and East Asia, including Japan, China, Vietnam, and Cambodia (d). Scale bar indicates nucleotide substitutions per site.

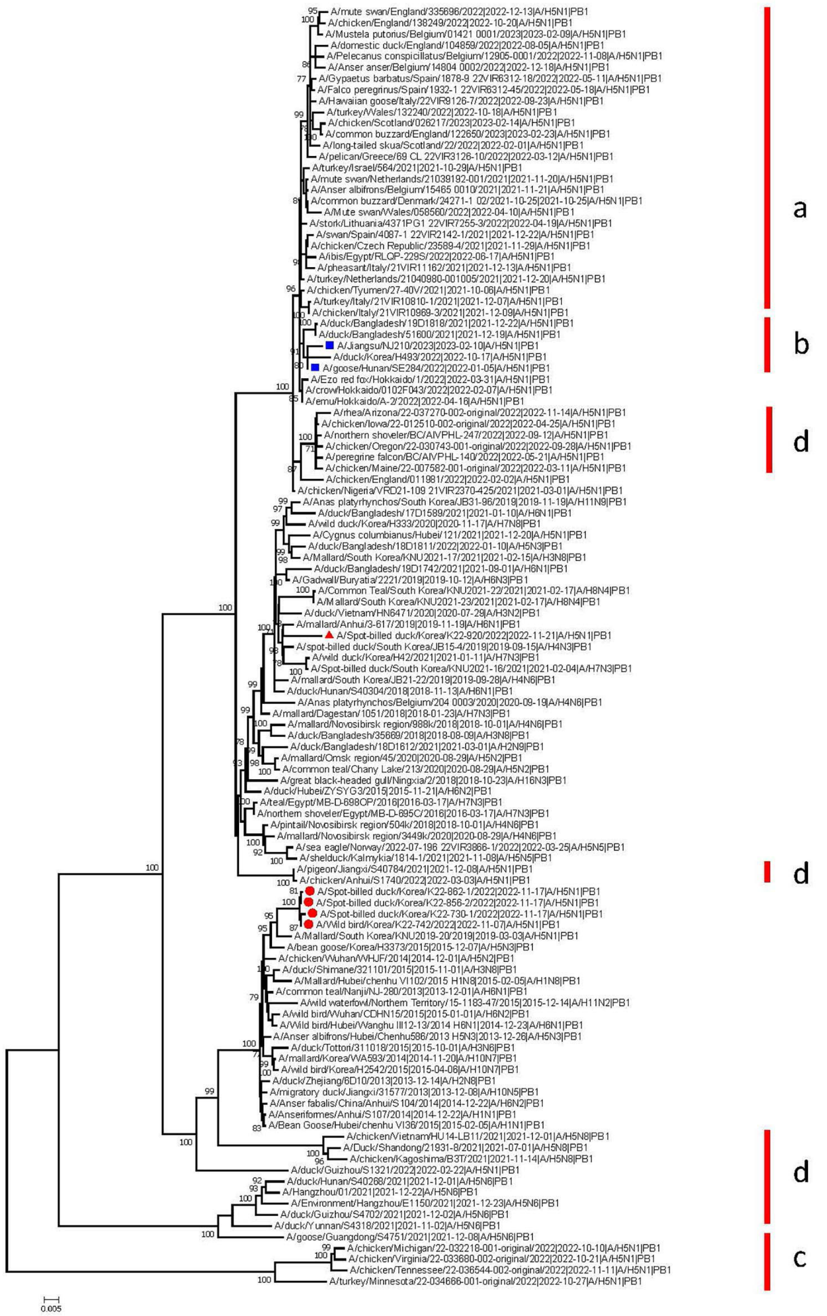

Appendix 1 Figure 2. Maximum-likelihood phylogenetic tree of polymerase basic 1 protein gene segment from avian influenza viruses. Bootstrap values >70% are shown. Highly pathogenic avian influenza virus isolates K22–862–1, K22–856–2, K22–742, K22–730–1 from South Korea are indicated by solid red circles, K22–920 from South Korea by solid red triangle, and genotype G10 isolates A/Hunan/SE284/2022 and A/Jiangsu/NJ10/2023 from China by blue rectangles. Red vertical lines indicate gene clusters mainly detected in Europe (a), Asia, including Bangladesh, Russia, and China (b), North America (c), and East Asia, including Japan, China, Vietnam, and Cambodia (d). Scale bar indicates nucleotide substitutions per site.

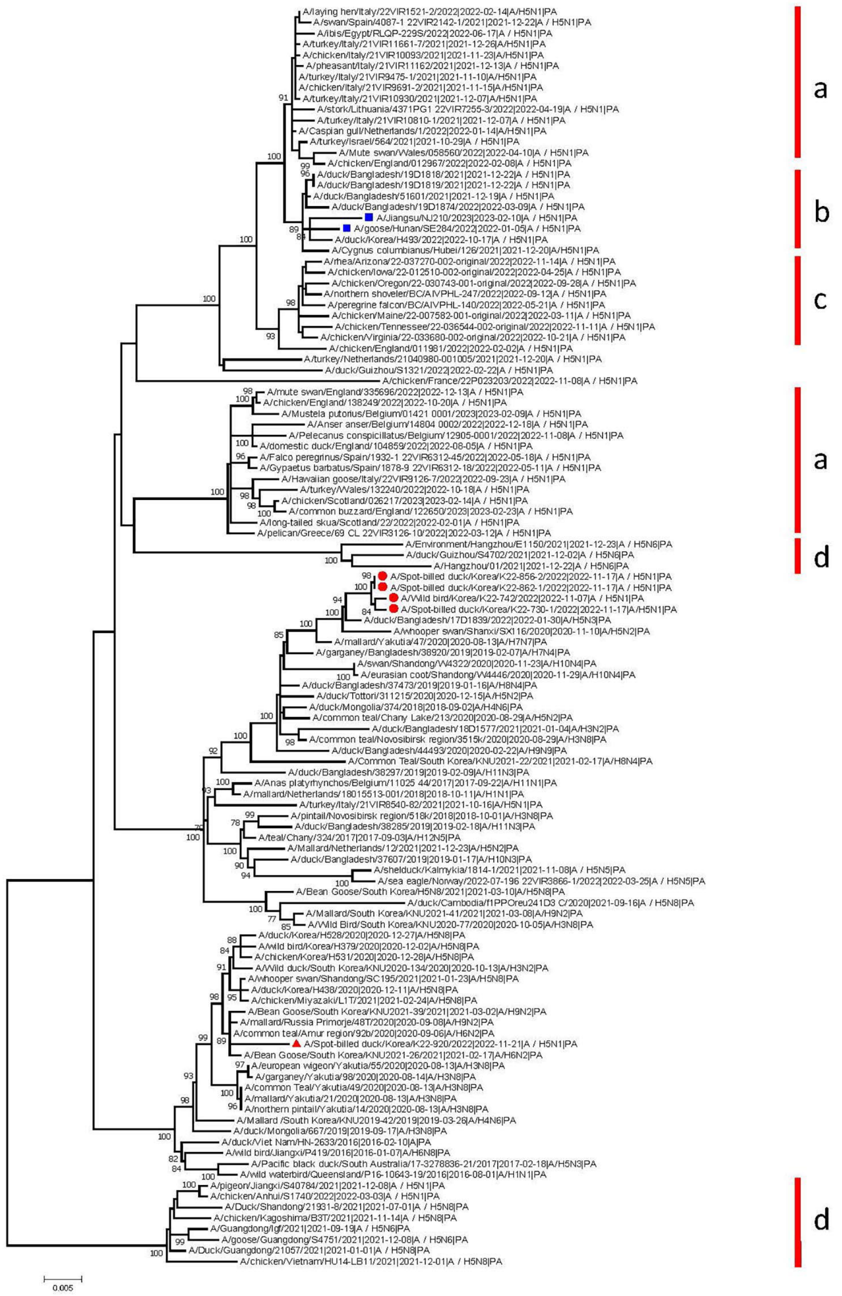

Appendix 1 Figure 3. Maximum-likelihood phylogenetic tree of polymerase acidic protein gene segment from avian influenza viruses. Bootstrap values >70% are shown. Highly pathogenic avian influenza virus isolates K22–862–1, K22–856–2, K22–742, K22–730–1 from South Korea are indicated by solid red circles, K22–920 from South Korea by solid red triangle, and genotype G10 isolates A/Hunan/SE284/2022 and A/Jiangsu/NJ10/2023 from China by blue rectangles. Red vertical lines indicate gene clusters mainly detected in Europe (a), Asia, including Bangladesh, Russia, and China (b), North America (c), and East Asia, including Japan, China, Vietnam, and Cambodia (d). Scale bar indicates nucleotide substitutions per site.

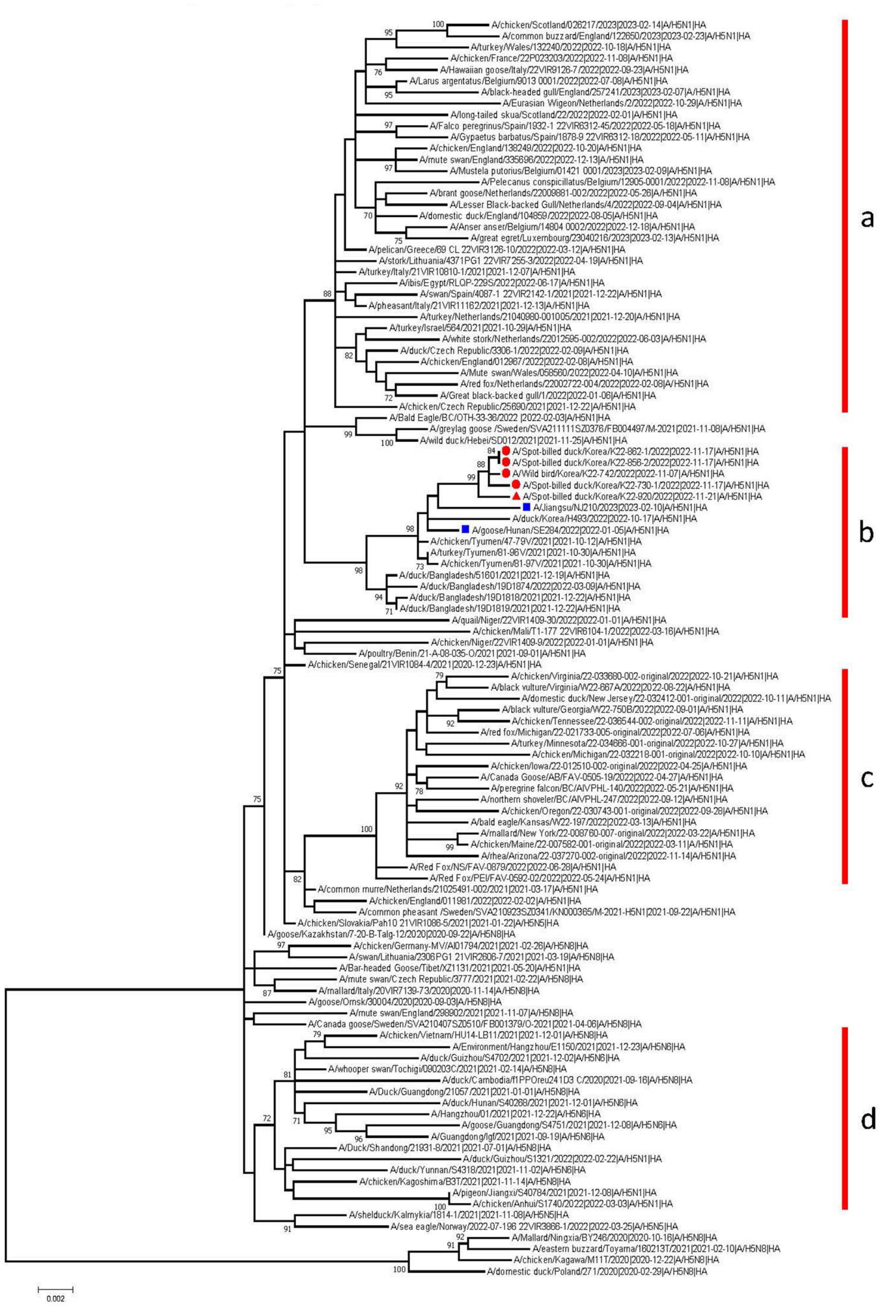

Appendix 1 Figure 4. Maximum-likelihood phylogenetic tree of hemagglutinin gene segment from avian influenza viruses. Bootstrap values >70% are shown. Highly pathogenic avian influenza virus isolates K22–862–1, K22–856–2, K22–742, K22–730–1 from South Korea are indicated by solid red circles, K22–920 from South Korea by solid red triangle, and genotype G10 isolates A/Hunan/SE284/2022 and A/Jiangsu/NJ10/2023 from China by blue rectangles. Red vertical lines indicate gene clusters mainly detected in Europe (a), Asia, including Bangladesh, Russia, and China (b), North America (c), and East Asia, including Japan, China, Vietnam, and Cambodia (d). Scale bar indicates nucleotide substitutions per site.

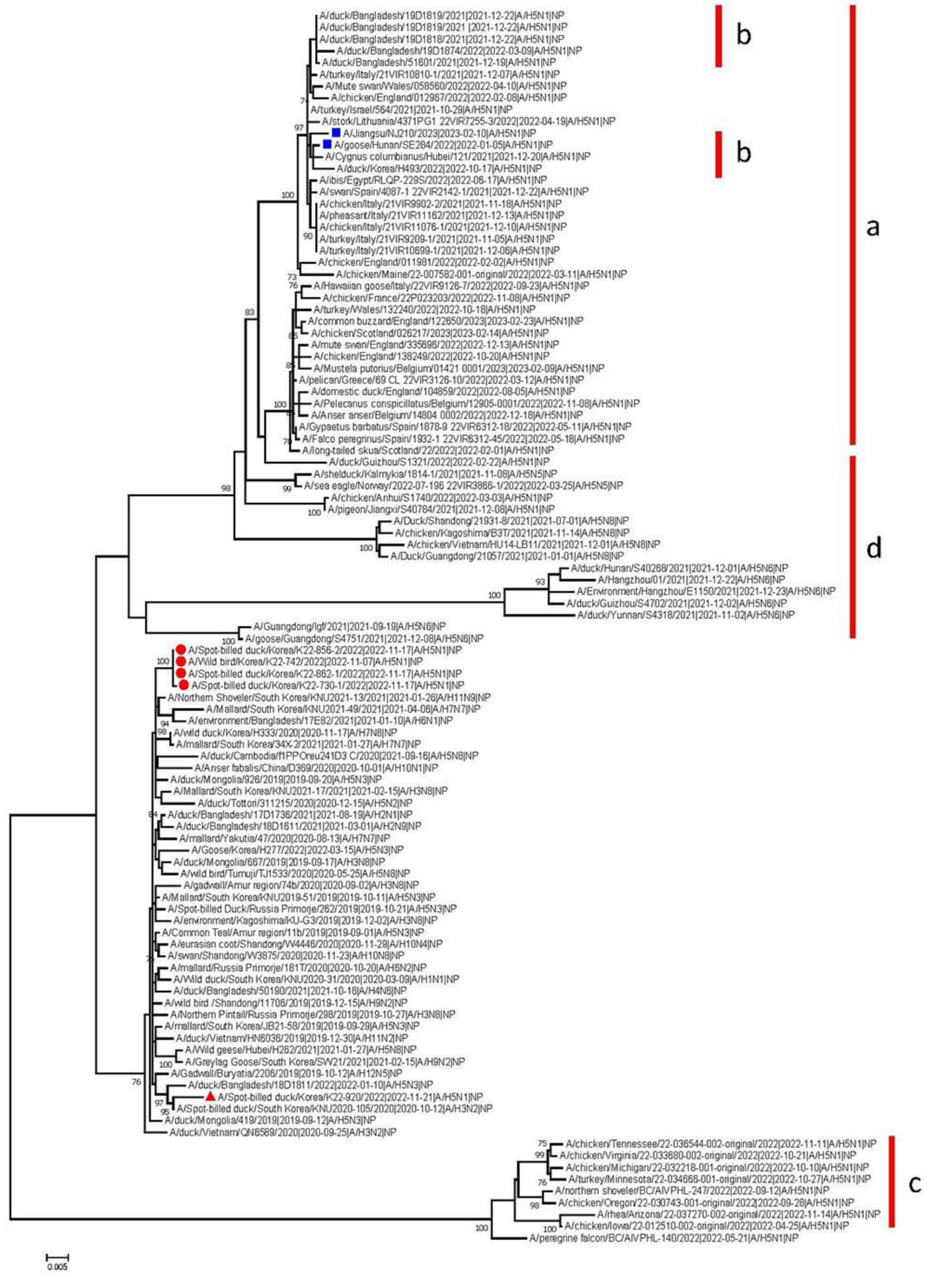

Appendix 1 Figure 5. Maximum-likelihood phylogenetic tree of nucleoprotein gene segment from avian influenza viruses. Bootstrap values >70% are shown. Highly pathogenic avian influenza virus isolates K22–862–1, K22–856–2, K22–742, K22–730–1 from South Korea are indicated by solid red circles, K22–920 from South Korea by solid red triangle, and genotype G10 isolates A/Hunan/SE284/2022 and A/Jiangsu/NJ10/2023 from China by blue rectangles. Red vertical lines indicate gene clusters mainly detected in Europe (a), Asia, including Bangladesh, Russia, and China (b), North America (c), and East Asia, including Japan, China, Vietnam, and Cambodia (d). Scale bar indicates nucleotide substitutions per site.

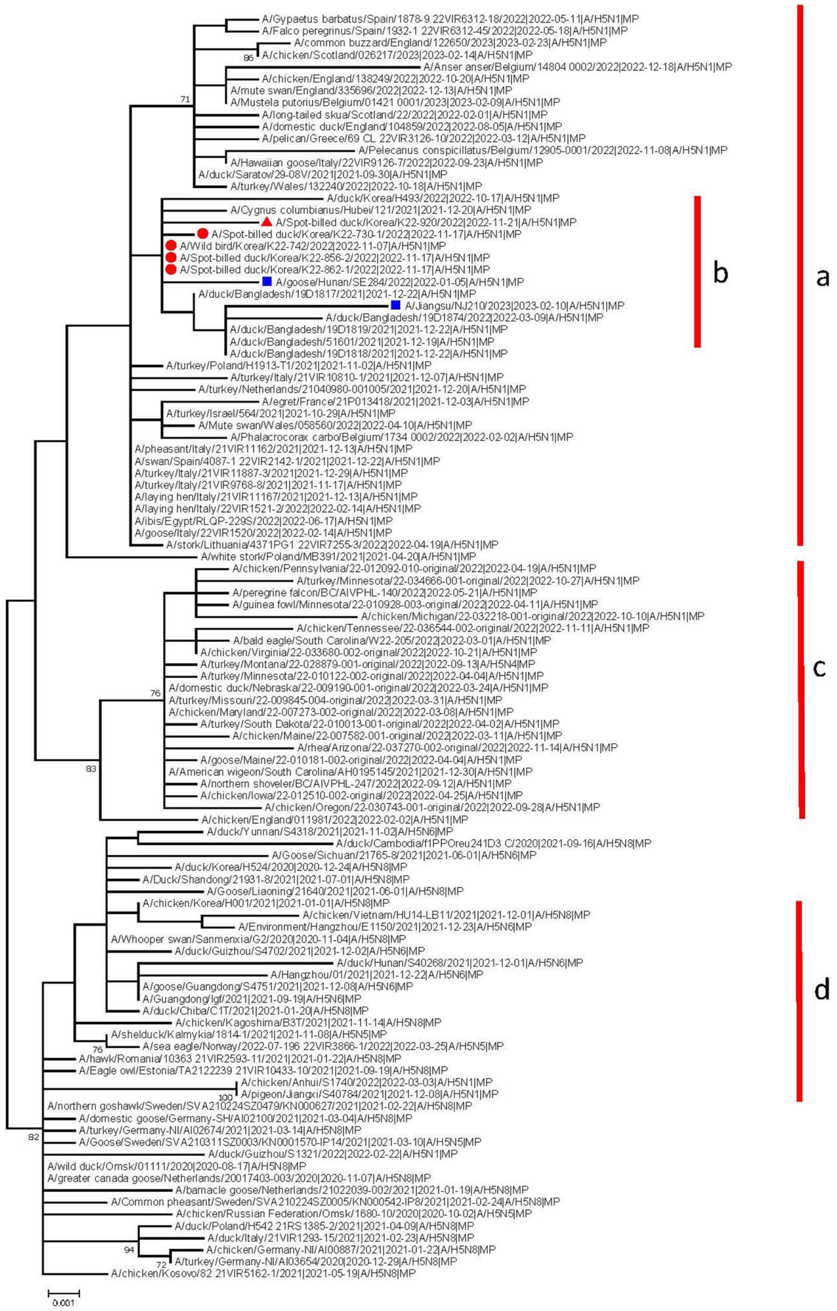

Appendix 1 Figure 6. Maximum-likelihood phylogenetic tree of neuraminidase gene segment from avian influenza viruses. Bootstrap values >70% are shown. Highly pathogenic avian influenza virus isolates K22–862–1, K22–856–2, K22–742, K22–730–1 from South Korea are indicated by solid red circles, K22–920 from South Korea by solid red triangle, and genotype G10 isolates A/Hunan/SE284/2022 and A/Jiangsu/NJ10/2023 from China by blue rectangles. Red vertical lines indicate gene clusters mainly detected in Europe (a), Asia, including Bangladesh, Russia, and China (b), North America (c), and East Asia, including Japan, China, Vietnam, and Cambodia (d). Scale bar indicates nucleotide substitutions per site.

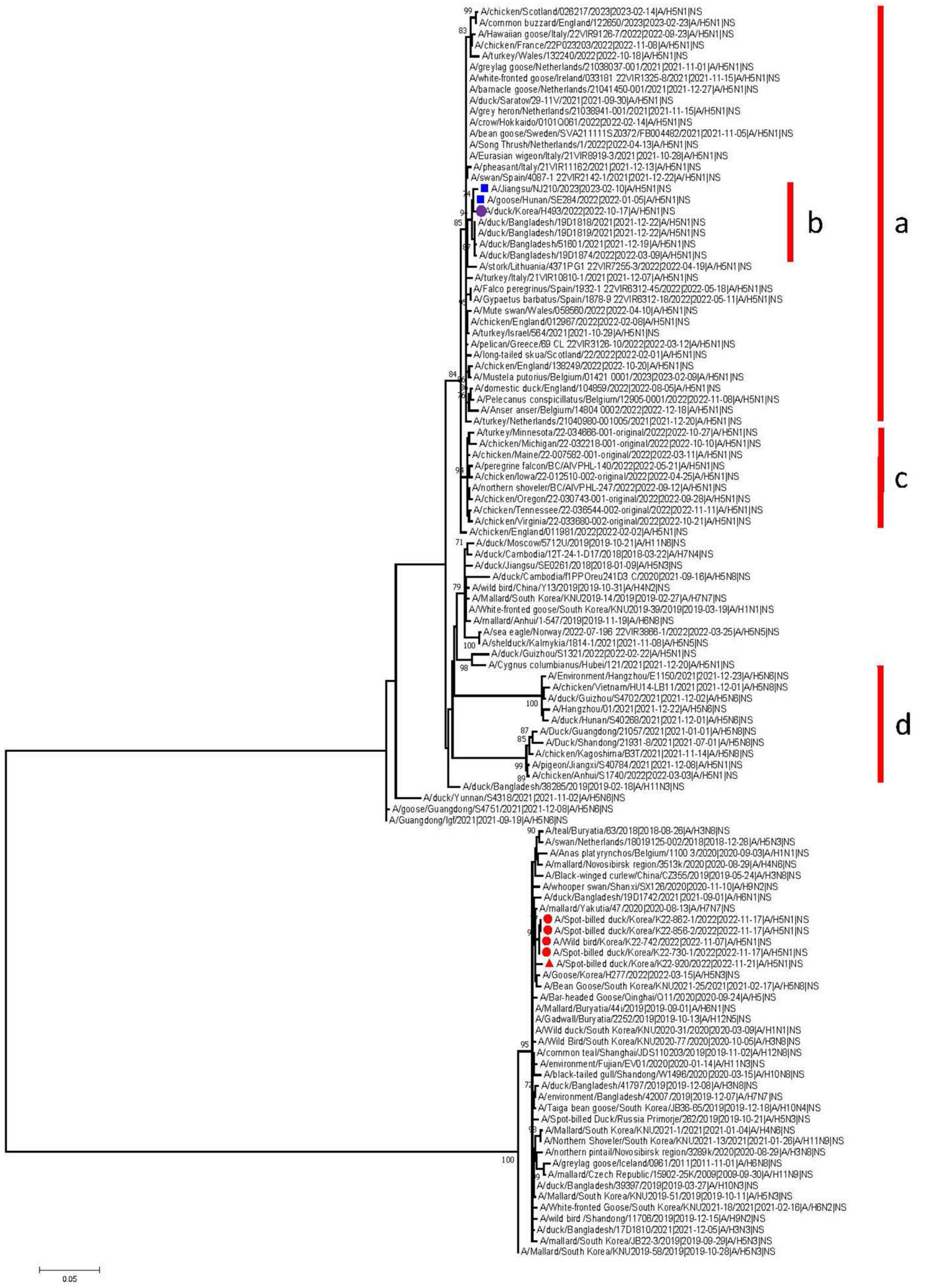

Appendix 1 Figure 7. Maximum-likelihood phylogenetic tree of matrix protein gene segment from avian influenza viruses. Bootstrap values >70% are shown. Highly pathogenic avian influenza virus isolates K22–862–1, K22–856–2, K22–742, K22–730–1 from South Korea are indicated by solid red circles, K22–920 from South Korea by solid red triangle, and genotype G10 isolates A/Hunan/SE284/2022 and A/Jiangsu/NJ10/2023 from China by blue rectangles. Red vertical lines indicate gene clusters mainly detected in Europe (a), Asia, including Bangladesh, Russia, and China (b), North America (c), and East Asia, including Japan, China, Vietnam, and Cambodia (d). Scale bar indicates nucleotide substitutions per site.

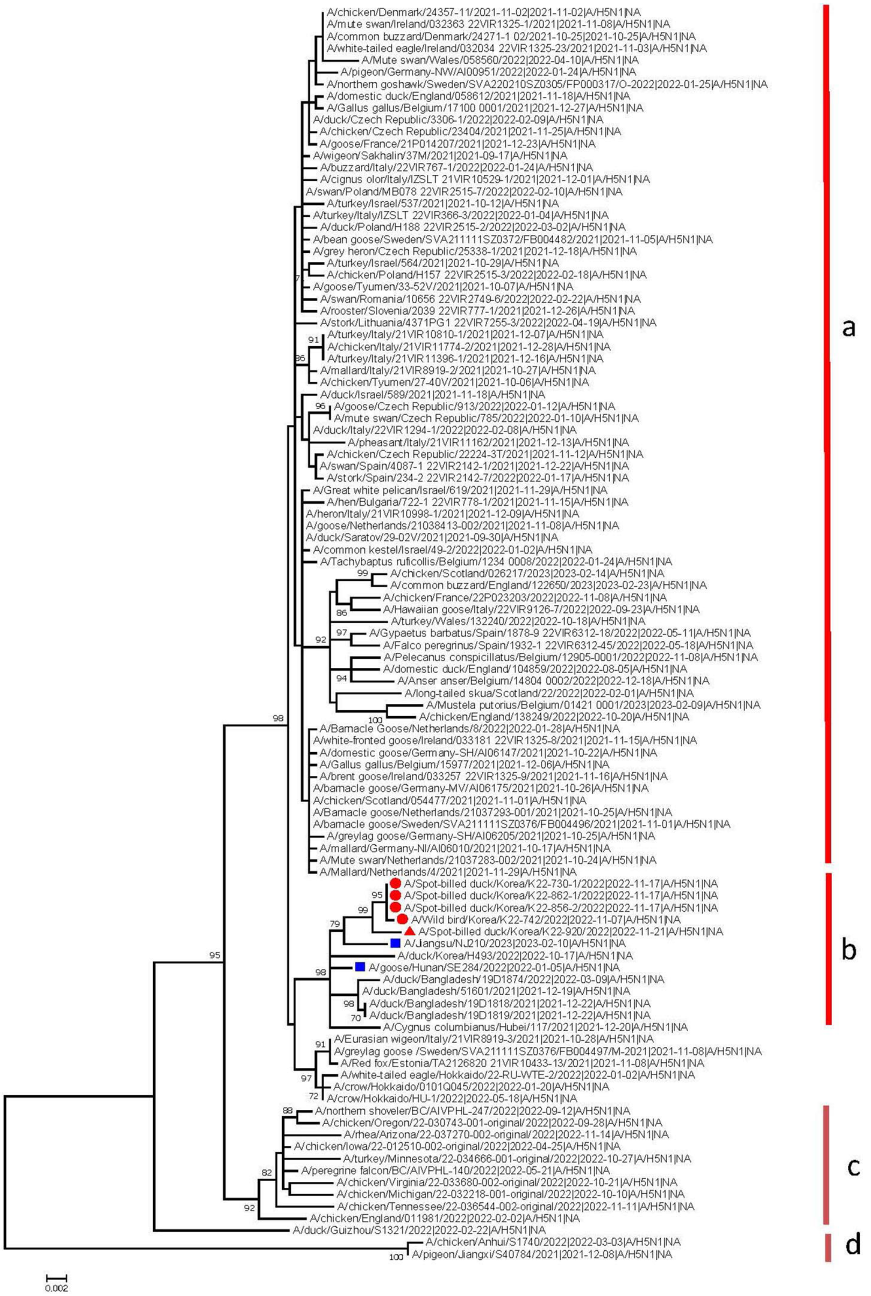

Appendix 1 Figure 8. Maximum-likelihood phylogenetic tree of nonstructural protein gene segment from avian influenza viruses. Bootstrap values >70% are shown. Highly pathogenic avian influenza virus isolates K22–862–1, K22–856–2, K22–742, K22–730–1 from South Korea are indicated by solid red circles, K22–920 from South Korea by solid red triangle, and genotype G10 isolates A/Hunan/SE284/2022 and A/Jiangsu/NJ10/2023 from China by blue rectangles. Red vertical lines indicate gene clusters mainly detected in Europe (a), Asia, including Bangladesh, Russia, and China (b), North America (c), and East Asia, including Japan, China, Vietnam, and Cambodia (d). Scale bar indicates nucleotide substitutions per site.

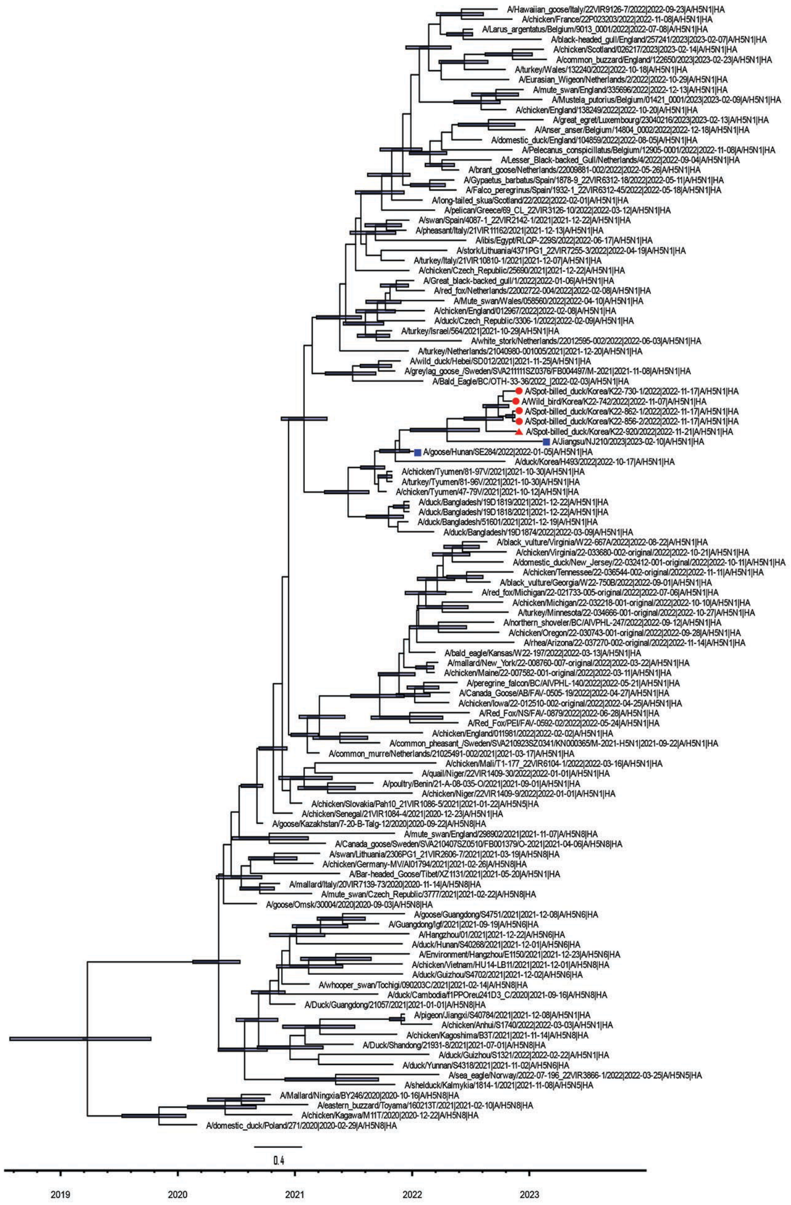

Appendix 1 Figure 9. Time-scaled maximum clade credibility phylogenetic tree of hemagglutinin gene segments of avian influenza viruses. Node bars represent 95% Bayesian credible intervals. The x-axis defines the time scale in decimal years. Highly pathogenic avian influenza virus isolates K22–862–1, K22–856–2, K22–742, K22–730–1 from South Korea are indicated by solid red circles, K22–920 from South Korea is indicated by solid red triangle, and genotype G10 isolates A/Hunan/SE284/2022 and A/Jiangsu/NJ10/2023 from are indicated by blue rectangles. Scale bar indicates nucleotide substitutions per site.
